# The Racer's Mind—How Core Perceptual-Cognitive Expertise Is Reflected in Deliberate Practice Procedures in Professional Motorsport

**DOI:** 10.3389/fpsyg.2018.01294

**Published:** 2018-08-13

**Authors:** Otto Lappi

**Affiliations:** ^1^Cognitive Science, Department of Digital Humanities and Helsinki Centre for Digital Humanities (Heldig), University of Helsinki, Helsinki, Finland; ^2^TRUlab, University of Helsinki, Helsinki, Finland

**Keywords:** expert performance, perceptual-cognitive expertise, deliberate practice, knowledge elicitation, qualitative methods, sport science, motor racing, driving

## Abstract

The exceptional performance of elite practitioners in domains like sports or chess is not a reflection of just exceptional general cognitive ability or innate sensorimotor superiority. Decades of research on expert performance has consistently shown that experts in all fields go to extraordinary lengths to acquire their perceptual-cognitive and motor abilities. Deliberate Practice (DP) refers to special (sub)tasks that are designed to give immediate and accurate feedback and performed repetitively with the explicit goal of improving performance. DP is generally agreed to be one of the key ingredients in acquisition of expertise (not necessarily the only one). Analyzing in detail the specific aspects of performance targeted by DP procedures may shed light on the underlying cognitive processes that support expert performance. Document analysis of professional coaching literature is one knowledge elicitation method that can be used in the early phases of inquiry to glean domain information about the skills experts in a field are required to develop. In this study this approach is applied to the domain of motor racing - specifically the perceptual-cognitive expertise enabling high-speed curve negotiation. A systematic review procedure is used to establish a corpus of texts covering the entire 60 years of professional motorsport textbooks. Descriptions of specific training procedures (that can be unambiguously interpreted as DP procedures) are extracted, and then analyzed within the hierarchical task analysis framework driver modeling. Hypotheses about the underlying cognitive processes are developed on the basis of this material. In the traditional psychological literature, steering and longitudinal control are typically considered “simple” reactive tracking tasks (model-free feedback control). The present findings suggest that—as in other forms expertise—expert level driving skill is in fact dependent on vast body of knowledge, and driven by top-down information. The knowledge elicitation in this study represents a first step toward a deeper psychological understanding of the complex cognitive underpinnings of expert performance in this domain.

## Introduction

“*Maslow (**1971**) if we want to know how fast a human being can run, then it is no use to average out the speed of a ‘good sample' of the population.”*

– Abraham Maslow (The Farther Reaches of Human Nature, p.7)

World-class athletes and experts in certain forms of art such as classical ballet allow us to observe human performance at the limit of physiological capability. Highly trained individuals capable of carrying out complex motor actions with such speed, precision and - when necessary - power that from the perspective of the recreational practitioner their performance may seem almost superhuman. In fields requiring less physical effort the perceptual-cognitive expertise is no less remarkable (for example, the world record for solving the Rubik cube currently stands at 4.59 s).

Yet research on the cognitive foundations of expertise, in a wide variety of domains, has over the past 35 years consistently shown that such excellence is not a reflection of vastly superior innate sensorimotor ability or general intelligence. Experts and non-experts have—more or less—similar general capacities for body movement and higher brain function. What is different is that experts go to extraordinary lengths to acquire knowledge and skills that make consistently superior expert performance possible (Ericsson, 2006; Kaufman and Duckworth, 2015; Ericsson et al., 2018). Clearly, a better understanding of the relevant cognitive processes and the general principles and specific mechanisms of underlying higher brain functions would be beneficial for understanding human development and learning, with applications in education, sports coaching and neurological rehabilitation.

Because of the very nature of expert performance—highly developed skill exhibited in complex real-world situations—studying expertise requires a multidisciplinary and multi-methods approach, combining among others experimental psychology, neuroscience, computational modeling and qualitative knowledge elicitation methodologies (Gobet, 2016; Ericsson et al., 2018). The approach in this paper is that analysis practice procedures themselves can give clues about the underlying perceptual-cognitive processes that are being targeted by the practice.

The domain of interest is the core perceptual-cognitive expertise in motorsport: the skill involved in operating a motor vehicle to negotiate bends on a track at the highest attainable speed. Motor racing is a highly developed field of professional sport where the competing athletes are required to make demanding perceptual-cognitive judgments under extreme time pressure (i.e. moving at very high speeds), and physiologically highly demanding conditions (Jacobs et al., 2002; Watkins, 2006; Potkanowicz and Mendel, 2013).

For understanding fundamental processes such as controlled interception of locomotor targets, avoidance of obstacle, locating and identifying relevant information and visuomotor coordination it is possible to develop theoretical models, and psychophysical and experimental psychology methods can be used to individually test the discriminability and learnability of putative cues. However, only careful analysis of natural behavior in its ecological context can determine which cues and strategies are actually used in the real world. Here, research on different sports has been instrumental in our gradually developing understanding of human perception and cognition “in the wild” (Regan and Gray, 2000; Land and Tatler, 2009).

The study of (expert) driving is in many ways an attractive model behavior for studying the cognitive basis of expertise:

1. The determinants of performance that limit a driver's speed are mostly information processing limits of the brain, not physical limits of the body. Performance is largely determined by perceptual-cognitive expertise, not the force or speed that the racing driver can move the controls, or the power that her body can generate to sustain locomotion.
2. Parameterizing the relevant stimulus environment and the behavioral patterns is relatively straightforward. The 3D geometry of race tracks is fairly simple, and even the highest level of expertise is displayed through a low–dimensional system (controllers with few degrees of freedom).
3. Driving is a fairly unique task in that it is possible to cover the full range of expertise from naïve to expert (e.g., driving school to professional racing driver). Whereas a common problem in the expert performance approach is it is difficult to find representative tasks that are at the same time doable for the novice but not trivial to the expert, the apparently simple act of negotiating a series of bends at maximum speed is just such a task. What is more, this task has a simple unambiguous and ecologically maximally valid performance measure: elapsed time.

Yet, while vehicle system dynamics engineering has achieved a detailed working understanding of the complex dynamics of the racing car (Milliken and Milliken, 1995), there is to date little empirical work on the even more complex dynamics of the sensory and motor physiology of the racing driver.

The physiological work (Schwaberger, 1987; Jacobs et al., 2002; Backman et al., 2005; Baur et al., 2006; Brearley and Finn, 2007; Schneiders et al., 2010; Filho et al., 2015) in the main focuses on cardiovascular stressors, and does not elucidate sensory physiology and motor coordination. Bernardi et al. (2013, 2014) used fMRI to study group differences in anatomy and task-dependent brain activity between expert racing drivers and normal controls. Specifically in (2013) they found smaller volume recruitment and stronger connections among task-related regions in (easy) motor reaction and visuomotor tasks for Formula 1 drivers compared to normal controls, which they interpreted in terms of higher “neural efficiency” (Haier et al., 1992; see also Poldrack, 2015; Vickers and Williams, 2017) in task organization. In (2014) they used inter-subject correlation (ISC) to show group level differences in brain activation between professional racing drivers and “naïve” controls when watching shown in-car footage of an F1 car driving on official circuits. Bilateral activation in visuomotor and medial temporal structures was observed in both groups. The racing drivers showed significantly more synchronous activation in the prefrontal cortex, cerebellum, caudate nucleus, posterior parietal and anterior cingulate and retrosplenial cortex. Voxel-based morphometry analysis showed higher gray matter density in a number of areas, including the thalamus, basal ganglia, inferior frontal and precentral cortex, and the retrosplenial region. Intriguingly, the individual variation in retrosplenial gray matter density was correlated with career success in the professional racing drivers (for theoretical interpretation and discussion relevant to the present study, see Lappi, 2015).

Research on the perceptual-cognitive basis of task performance in this domain—which would give a more solid foundation for interpreting brain imaging data—is virtually non-existent. Land and Tatler (2001) used eye tracking to record the visual behavior of one racing driver on a circuit (van Leeuwen et al., 2017, replicated their study in a fixed-base simulator with a larger number of participants, including both experts and novices). The main findings of this work were that (i) racing drivers visually “anticipate” upcoming bends—although both the mechanisms underlying this anticipatory behavior and the specific visual target(s) remain speculative, and (ii) a clear dependency between head rotation and subsequent car rotation—a “steering with the head strategy” (Land and Tatler, 2001)^1^. Mondada (2018) used video-based qualitative interaction analysis to investigate coaching on driving technique of an individual driver on a race track, focusing on the sequential and indexical character of the interaction patterns, But as this was a case study, few general conclusions can be drawn.

## Rationale and aims of the study

Qualitative methods based on *knowledge elicitation* by expert interviews and documentation analysis can be useful for identifying recurring ecologically representative task elements and piecing together a full picture of the perceptual-cognitive expertise, especially in early phases of inquiry (Hoffman et al., 1995). Document analysis, in particular, may have value in bringing accumulated knowledge into the scientific domain (Bowen, 2009; for its use in sports science see e.g., Smith et al., 2018). An adequate task analysis, grounded in existing domain expert knowledge gleaned by qualitative methods, will be useful to understand actual real-life expert performance and to design informative and ecologically representative experiments^2^.

In mature fields many essential practice procedures involved in the acquisition of expertise become codified in fairly standard linguistic and diagrammatic form in textbooks and training manuals. Beginning with Taruffi (1959), the knowledge and thought processes involved in racing driving have been analyzed in increasing detail, codifying and extending the domain knowledge in the field. There is thus readily available 60 years' worth of literature on the practice procedures in motorsport used for developing expert driving skill. While deriving task performance insight from such material lacks the spatial and temporal detail of direct observation and measurement of actual performance, the advantage is that the description will be grounded in the concepts and practices of the field experts and, as a knowledge elicitation method it may provide a more balanced and systematic basis for developing practice-informed hypotheses than anecdotal observation or interviewing individual expert informants.

The aim of this project was to connect the training material through some coding framework (for which DP eventually was chosen), to driver modeling literature: the McRuer et al. (1977) framework. This is the theoretical framework the author has been working on, and which is the conceptual basis of driver-in-the-loop computational modeling in motorsport engineering, sharing conceptual and historical roots with driver models in experimental psychology (for review see Lappi and Mole, 2018).

A systematic search and content extraction based on an operational definition of DP (DP1-DP4, below, grounded in the psychological literature) was used to establish a set of primary material. This material depicts Deliberate Practice Procedures (DPP) in motor sport. Their likely target mechanisms were interpreted in terms of putative core perceptual-cognitive mechanisms, organized within (an extension) of the conceptual framework of McRuer et al. (1977). These integrative hypotheses are meant to represent some but not all core perceptual and cognitive processes underpinning expert skill in the domain. To anticipate the findings presented in the Results and Discussion sections, it is perhaps remarkable how the cognitive mechanisms underlying expert performance in this domain differ, quite radically in some respects, from everyday driving and typical laboratory steering tasks.

## Materials and methods

Qualitative document analysis was used to glean information on the skills experts are expected to develop. The methodological approach can be considered a variant of Grounded Theory (GT). GT has been used in numerous studies in sports science to develop domain insight into a wide number sports including work that aims to understand skill and expert cognition (Eccles et al., 2002; MacNamara et al., 2010; for discussion of GT in to sports science, see Weed, 2009, 2010; Holt and Tamminen, 2010a,b). More detailed exposition and methodological reflection on the steps taken, the choices made, and their rationales are given in Supplementary Methods (https://doi.org/10.6084/m9.figshare.6365441).

**Figure 1:**
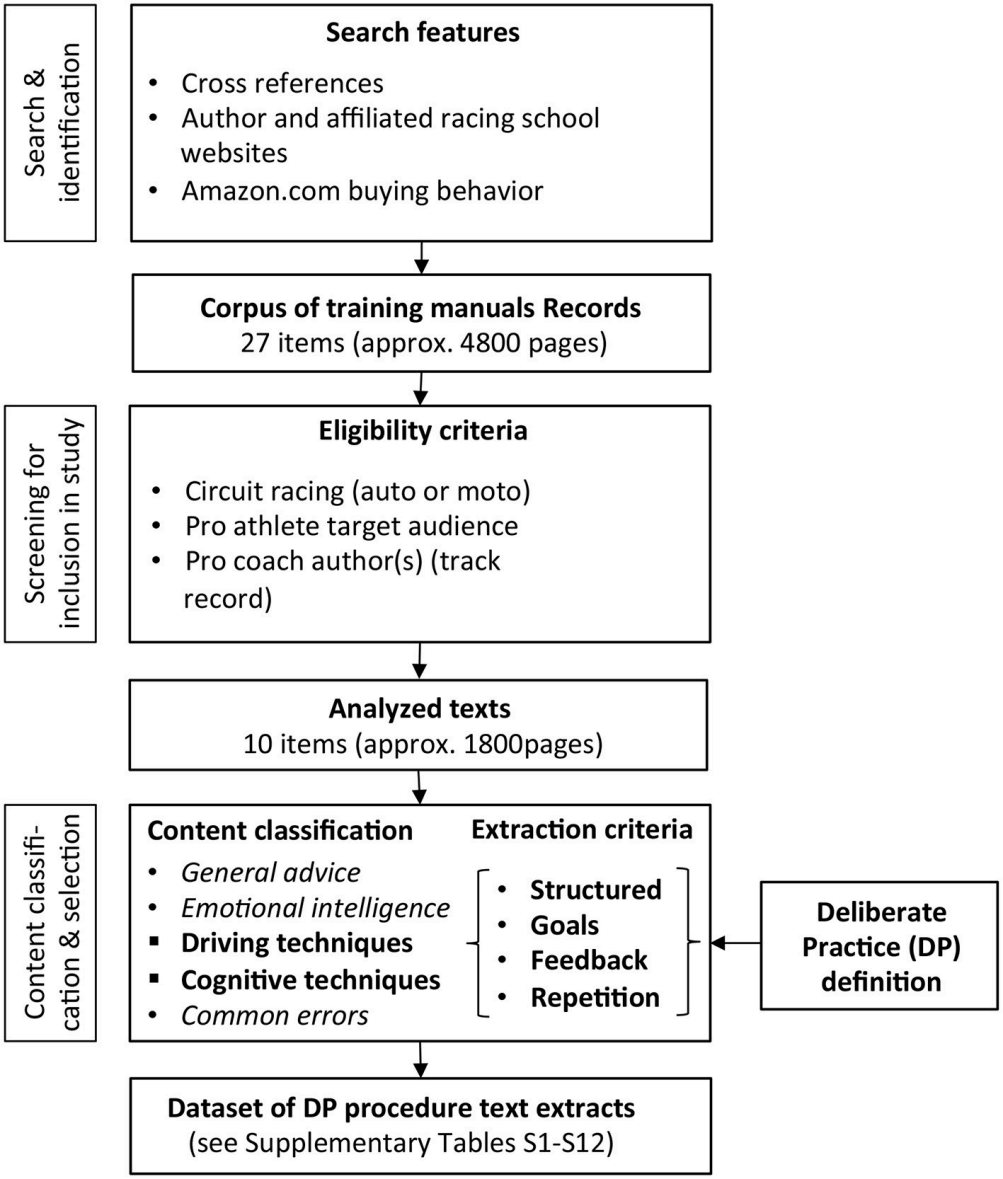
Design of the systematic thematic analysis. This text data collection and content extraction scheme was employed to conduct a search of the literature, select part of it for inclusion in the study, and finally to form the final corpus and classify it.

The logic of the study is as follows: The first step was gathering a representative corpus of material for content extraction and analysis. A systematic procedure (Figure 1) is used to establish from this diverse literature a more focused corpus for analysis. From this, a dataset of descriptions of training procedures that qualify as Deliberate Practice (DP) is identified and extracted (in other words, DP framework is used to codify and systematize the data). Note that the literature in does not represent academic research, so the aim of using a PRISMA-type procedure was not to establish a balanced assessment of a consensual view or argue for theoretical conclusions (as in a systematic review paper)—but to develop the corpus in a reproducible way.

### Search, inclusion criteria and content extraction

The aim was comprehensive coverage of books published on professional racing techniques written from the racing driver's point of view (that is, driver training as opposed to vehicle design, engineering and tuning material). Both auto racing and motorcycle racing books were included, as many of the task demands and techniques appear to be similar. To keep the number of items manageable the decision was made to exclude other forms of motorsport (e.g., karting, rallying, autocross & motocross, drag racing…), books on non-professional “track day” circuit driving, as well as books on “performance driving” skills on public roads. (Auto)biographies, material in magazines, leaflets published in Kindle form, and material on websites discussion forums was not included.

As the literature is not indexed in academic publication databases, cross-references between the books, author and racing school websites, and Amazon buying behavior links were used iteratively. A total of 28 items were obtained (Table 1), spanning nearly sixty years and comprising of a total of about 4,800 pages.

**Table 1:** The entire corpus of motorsport training manuals used in this study.

**Item**	**Year**	**References**	**Pages**	**Auto or moto**	**Incl**.
1	1959	Taruffi, 1959	126	Auto	no
2	1959	Jenkinson, 1959	222	Both	no
3	1963	Frère, 1963	138	Auto	no
4	1971	Johnson, 1971	143	Auto	no
5	1977	Lauda, 1977	245	Auto	no
6	1982	Holbert et al., 1982	109	Auto	no
7	1983	Code, 1983	114	Moto	yes
8	1986	Code, 1986	166	Moto	yes
9	1987	Fittipaldi and Kirby, 1987	136	Auto	no
10	1988	Roberts, 1988	217	Moto	no
11	1990	Prost and Rousselot, 1990	192	Auto	no
12	1993	Senna, 1993	208	Auto	no
13	1993	Anderson, 1993	191	Auto	no
14	1993	Code, 1993	115	Moto	yes
15	1996	Smith, 1996	190	Auto	no
16	1997	Lopez, 1997	277	Auto	yes
17	1998	Bondurant and Blakemore, 1998	140	Auto	yes
18	1998	Bentley, 1998	159	Auto	yes
19	2000	Bentley and Langford, 2000	151	Auto	yes
20	2003	Bentley, 2003	158	Auto	yes
21	2008	Castle, 2008	199	Auto	no
22	2009	Ibbott, 2009	184	Moto	yes
23	2011	Hornsey, 2011	118	Auto	no
24	2011	Bentley, 2011	332	Auto	yes
25	2015	Krumm, 2015	189	Auto	no
26	2016	Brouillard, 2016a	107	Auto	no
27	2016	Brouillard, 2016b	133	Auto	no
28	2016	Brouillard, 2016c	113	Auto	no

At this stage the literature was narrowed down further. The basis for the inclusion criteria was decided upon reading through the material, at which point it appeared most of it would fall quite naturally into two types of books: (i) those written by ex-drivers, often elite drivers with a world-class track record working with a journalist or a ghost writer, (ii) those written by professional coaches as study materials for their customers taking part in a racing school course or individual coaching (typically published in conjunction with a racing school operating at a racing track or purpose built facility). The ex-driver works generally seemed to contain a larger number of personal experiences, tips, ideas and ways of approaching different aspects of the profession. In contrast, the coaching literature, additionally, contained quite precise descriptions of technique, and procedures for working out solutions to typical problems. As the focus of this paper is to gain, through analysis of practice procedures. a detailed insight into the perceptual-cognitive basis of expert technique, it was decided to select for content analysis items that written by professional driving coaches. Once a content extraction and classification scheme would be developed on basis of this material, it would be relatively straightforward to extend it to category ii. books or even the parts of the literature left out from the search. This choice left ten items (four on motor cycle racing and six on auto racing, see Table 1), keeping the volume of literature manageable for manual content extraction.

The material was read through carefully, page by page, identifying individual passages that dealt directly with driving technique. Passages clearly dealing with perception, memory, decision making, problem solving, or other processes likely to be indicative of “core perceptual-cognitive expertise” were highlighted, focusing on passages that would contain enough concrete detail on procedures to be informative in view of developing a theoretical understanding of the task demands, and the skills and knowledge targeted by the practice (for more detailed explanation of this stage of content analysis, with examples of both excluded and included material, see Supplementary Methods).

It was at this stage that the DP framework was chosen as the codification framework. Other approaches considered at different stages of analysis included ABC “toolbox” of heuristics (Raab, 2012; de Oliveira et al., 2014), analysis of stereotypical error patterns in routine behavior (Cooper and Shallice, 2000), and the Generic Error Modeling System (Reason, 1990).

Typical (beginner) errors to avoid was a common theme in all the literature. However, many of the individual descriptions of the errors and correct techniques could be quite short, the intended interpretation requiring referencing explanatory material elsewhere in the book. This would have meant that to produce analyzable content extracts much more (re)organizing work and interpretive insight would have been needed. Also, the number of different errors would also have been prohibitively large, and, further, to make them more interpretable they would need to be contrasted with the corresponding correct techniques, enlarging the material to be analyzed still further.

In contrast, it appeared that passages describing “drills” or “exercises” would be more self-contained, and would have sufficient content and explicit grammatical structure to be meaningfully analyzed in terms of implied technique or even cognitive processes. It also turned out they could be unambiguously identified as DP, based on a definable set of criteria developed on basis of the academic literature on DP (see Supplementary Methods, and the next section). Indeed, as there is theoretical tension and debate in the literature as to when training can be legitimately considered DP (Ericsson, 2016), it seemed developing such criteria for the present work could be a useful contribution to the DP literature more generally. Passages fulfilling these criteria were also sufficiently small in number to present as a corpus for independent evaluation of the coding scheme. It was deemed that DP framework would be a good choice for this exploratory phase, to bootstrap domain insight (before perhaps tackling the more challenging content in follow-up work).

#### Operationalization of deliberate practice procedures (DPP)

In order to *systematically* apply the DP concept to a text corpus—and to do it more rigorously than in most of the literature, where, unfortunately, any reported training may be taken as indication of DP (cf. Ericsson, 2016)—a fairly precise operational definition is needed. The aim was a coding scheme that would be generalizable to other domains, not dependent on technical concepts or deep domain insight. This section describes the operational definition used here, and explains the theoretical rationale for it.

It is generally agreed that to attain world-class expertise it is necessary (but not necessarily sufficient Hambrick et al., 2014; Lombardo and Deaner, 2014) to engage in a large amount of diligent and well-designed practice. Presently, the most developed and established model of the acquisition of expertise is the Deliberate Practice (DP) Framework (Ericsson et al., 1993; Ericsson and Lehmann, 1996; Ericsson, 2006; for review of DP in sports see Baker and Young, 2014).

DP refers to practice (often solitary) assigned (often by a coach), where the primary motivation is improvement of a specific component of skill. It is not necessarily inherently enjoyable or rewarding, and the primary aim is not maximal performance, like in competition. In music training (Ericsson et al., 1993) it refers to concretely practicing technical or artistic aspects of performance, and is not meant to include studying music theory, public performances, or “jamming.” In chess, studying and determining best moves in mid game positions (and comparing one's choices to the choices of superior players) would count as DP, while playing in competition or time spent on just reading chess literature generally would not. Analogously, in sports one should not count as DP competition (unique events requiring maximal performance), general fitness training, or studying “the theory” of the sport.

The central assumptions of the DP framework are that (i) the level of performance an individual attains in a domain is monotonically related to the amount of deliberate practice they have accumulated, and that (ii) the attained level of expertise at the highest levels performance depends mainly on time invested in DP over the career (rather than total amount of domain experience generally, general cognitive ability, or innate domain specific “talent”). The often cited, often contested, and occasionally misinterpreted “10,000 h/10 year rule” says, further, that for elite levels of expertise in many domains, accumulation of experience on the order of 10,000 h of is needed. This would translate to 4 h/day over 10 years. These figures can give a useful ballpark approximation, but should be interpreted as indicating order of magnitude, rather than an exact figure (1,000 h or 1 year is generally not sufficient to attain true expertise, and 100 000 h or 100 years are obviously not necessary).

The essential theoretical import of the DP framework, however, is not the *amount* of practice *per se*. Some domains are presumably more complex or more developed than others in terms of complexity of accumulated technique, and level of competition, so that success in them depends on more finely tuned perceptual-cognitive skills (the traditional examples of chess and classical music are probably in the more extreme end of cognitive requirements). Also individual differences and training methods could make a difference to the speed of acquisition. Hence, 10 000 h as a strict universal rule would make little sense. Instead, the key contribution of DP framework is to distinguish between the *type* of domain experience that leads to the development of expertise and the type of experience that does *not*^3^.

Here we define a Deliberate Practice Procedure (DPP) as an explicit (e.g., verbal or pictorial) training task design that fulfills the following four characteristics (based on the core features of DP, as discussed in Ericsson et al., 1993; Ericsson and Lehmann, 1996):

*DP1. Structured activity*. DP is not “spontaneous” trial and error, but consists of activities designed, by the performer or a coach, to practice specific skills. The task may not be fully representative of the conditions of maximal performance, but may be simplified, restricted or modified in a way that isolates a specific subtask, and/or facilitates the diagnosis common errors and explicit monitoring of progress. To count as a DPP there should be clear task instruction, perhaps even a step-by-step walkthrough. (This in addition to what to aim for (cf. DP2), and what relevant feedback to pay attention to (cf. DP3). A progression of subtasks (cf. DP4) of increasing difficulty and complexity may be involved, designed to elicit progressively higher challenge, more subtle feedback, and to facilitate acquisition of relevant sub-skills (and to avoid or unlearn “bad habits”).
*DP2. Goal oriented*. The ultimate goal (motivation) for DP is self-improvement (so it need not be as inherently motivating as competition or achieving maximal performance). Thus, DP is not only rote repetition—the trainee needs to be “pushing the envelope” of performance. The proximate goal of a DPP is to be able to perform at a higher level of ability than previously a specific subtask or skill that can be practiced in isolation.
*DP3. Clear feedback*. Outcomes of action are immediate, and most importantly unambiguously informative (in terms of the attainment of specified proximal goal or the type and/or magnitude of error—not just that some error has occurred). This is by design (DP1), and the DPP should indicate what the relevant feedback is, and how it should be interpreted, i.e., specify what to do, or how to correct behavior. Full cognitive concentration on performance—a high level of situational awareness—is typically required for effective monitoring of feedback (this may be external or internal focus of attention as appropriate). Note, however, that the feedback need not be fully understandable to the trainee at a *reflective* level of explicit knowledge (i.e., the trainee need not be able to reason about causes and effects at a conscious level, at least not without further theoretical study and development of more refined mental models of action-outcome contingencies).
*DP4. Repetition*. DP consists in sustained engagement in repetitive drills. The concept of DP includes the requirement of high training volumes, rather than (just) high intensity. This implies motivation and willingness to expend effort (as DP may not always be inherently motivating like competition or play).

Deliberate practice—defined strictly, as here using the criteria DP1-DP4—is distinct from everyday experience, spontaneous play, or competition. DP differs from most forms of everyday experience which are not set up to maximize feedback, and where goals are usually implicit and vague. Like everyday activities, however, DP involves repetitive, routine-like behaviors, which makes DP different from competition, which requires summoning up maximal performance in a singular event. Spontaneous play and (non-serious) competition, on the other hand, are inherently motivating. They do not involve deliberately defined subgoals, nor do they require substantial motivation and self-discipline to sustain engagement in the long term, which DP does. Also, in everyday activities and spontaneous play the goal is not improvement *per se*, whereas in DP procedures (“drills”) are explicitly designed, and engaged in, to extend one's capability, often in a specific sub-skill.

Going again through the material page by page, passages were selected and extracted on the basis that they should conform to the strict definition of DP derived from the research literature. Specifically, it was required that in a “drill” (DP procedure) described in each individual extract it should be possible to identify all the characteristics (DP1 Structured, DP2 Goal oriented, DP3 Clear feedback, DP4 Repetition), explicitly stated or clearly implied from the context. This produced the final dataset of 12 passages were deemed to contain sufficiently unambiguously the content classification (DP1-DP4). These extracts comprise a small corpus of just over 3,200 words, which condenses specific information from the original several thousands of pages of material. The complete extracts and the content classification (see below) are given in Supplementary Results as Supplementary Tables S1–S12 (https://doi.org/10.6084/m9.figshare.6078632).

## Results

We next indicate how the operational DP definition outlined in the Methods fits to the material, which is the basis for considering the passages as instances of DP. Table 2 shows selected DPP classified extracts. Please note that to anticipate the Discussion, they are arranged “bottom up”—from sensory “feel” to use of symbolic map representations—so that it will be easier to compare Tables 2, 3 in the Discussion.

**Table 2:** Example passages from the twelve extracted Deliberate Practice Procedures (DPP), analyzed into DP1-DP4 operationalizing DP. For the complete dataset of full extracts please see Supplementary Results: https://doi.org/10.6084/m9.figshare.6078632.

**Procedure**	**DP1: Structure**	**DP2: Goals**	**DP3: Feedback**	**DP4: Repetition**
1. “Feel” for grip and traction	“coaching” “exercise” “exercise” “forgetting … lap times practice” “put a 1 to 10 rating scale on it” “as you drive around the track, you can actually call out the amount of traction”	“improvement” “reading how much traction the tires have around every inch of the track”	“focusing on sensing the tires' traction” “make note of the vibrations and feedback through the steering wheel … lighter or heavier” “make note of the sound coming from the tires … more or less noise” “how does the car feel” “how much warning do the tires give”	“traction sensing sessions” “dedicate all or part of a practice session”
2. Sense of speed #1	“practice” “method” “exercises” [step-by-step]	“estimating speed, based solely on sensory input and not on the speedometer” “very accurate and most important consistent at judging and establishing a specified speed”	“see how accurate you are” “check how well you did”	“do it again and again” “over and over again”
3. Sense of speed #2	“technique” “using a pylon or pavement marker as a reference point” “your assistant should then ask you to increase your corner entry speed by 2 miles per h” “try 1 mile per hour less” [step-by-step]	“goal of entering the turn at exactly the same speed” “goal … is to consistently be at the same speed as you turn in to the corner” “to enter every corner on a race track at the same speed … within 1 mile per h” “calibrate your speed sensing with reality”	“radio to you the speed the speed you were traveling as you turned into the corner” “corner speed varies more than 1 mile per h” “See if you know what that small increase feels like” “how does that feel?”	“10 laps” “for at least 10 laps in a row”
4. Sense of speed #3 and speed adjustment	“drill” “drill”	“turn at the right speed … your target speed” “awareness of speed”	“your entry speed was right”	“first … second … third … ways to do”
5. “Smooth” control	“practice” “practice” “practice” “think of the word 'squeeze'; think of the word 'ease”'	“squeeze [on the gaze pedal],” “ease [off the gaze pedal] gently,” “squeeze [on the brakes] smoothly and progressively,” “feed in the required steering input,” “place [the shifter] in gear…with finesse” “squeeze the brakes on smoothly, firmly and progressively,” “release the brake pedal very gently”	“Don't pounce on the gas pedal,” “Don't slam on the brakes,” “Don't yank or jerk the steering wheel,” “so that you don't actually feel the point at which the brakes are fully released,” “so that you can't feel the exact point where the car comes to a complete stop”	“everyday driving,” “when driving on the street,” “every day on the street,” “becomes second nature or habit,” “on the street,” “do it enough on the street”
6. “Looking ahead”	“It takes practice” “begin practicing” “help you determine”	“looking farther ahead than you do now” “where to look” “look far enough ahead … but not [too] far” “With [reference points] you have a choice of where to look” “Having enough [reference points] … you can see enough to keep the track ‘opened up”'	“accelerating the scene” “you lose your feel for where you are on the track” “opening up the track, making it appear larger. When you look to far or too close … the track seems to narrow.” “your signal to either change [reference points] or to find more of them … Adjust the [reference points] so the scene is moving at the right speed for you”	“on the street”
7. Situational awareness #1	“work on seeing” “train yourself”	“be aware of everything and everyone around you” “be very focused, and yet be able to notice other things around you” “being aware of everything around you” “to know what's going on around you” “anticipate what they are going to do”	“make note of all the other cars around you – especially the ones you can't see directly in the mirrors” “keep track of cars behind and beside you”	“practice this on the street” “practice on the street” “at all times”
8. Situational awareness #2	“practice” “practicing” “practicing” “ask your brain” “ask your brain”	“be aware of everything along the side of the roadway” “allow your brain to take in more information” “aware of everything around you” “aware of other cars around you on the track—without having to put much, if any, concentration in it”	“Make note of the ground and the grass and the trees in great detail.” “note the colors, the type and amount of leaves on the rtees, the condition of the bark, whether the ground is made up of mostly dirt or rocks etc., the speed at which they pass by”	“while driving in the steet, and also in all other activities in your life.” “in your everyday world” “in traffic on the street” “the more you practice this…”
9. “Wide–screen” peripheral vision	“drill” “practice” “practice” [step-by-step]	“the correct seeing techniques” “moving your attention around, while looking at one spot or area”	“aware of other areas”	“as you're driving to the races or just sitting in a chair” “can take time to develop”
10. Finding the correct apex	“tool” “technique” “late apex at first” “When you feel you should turn in, overrule your instinct and turn later. Aim for an apex point that's later than you expect it eventually will be” “start with a late apex, then begin turning slightly earlier”	“find the right line” “determining whether you had the correct apex” “able to stay just barely on the track at the exit, while accelerating as hard as possible” ”the car will naturally want to follow a path out to the exit point“	“see what happens at the exit” “Look for an RPM improvement at the track-out. If the exit speed is improving, keep moving the turn-in earlier until symptoms of the early apex start to show up.” ”you come out of the corner having to turn more to keep from running off the road“ “not using all the road on exit” “too close to the inside corner”	
11. Visuali–zation and timing	[Step-by-step] “Close your eyes and think of a race track” “timing your memory”	“go through it exactly as fast as the last time you rode there” “enough reference points” “having sufficient [reference points]” “a better sense of time because … you have points to mark your motion around the track”	“much too long or much too short”	(implied; you need to develop reference points for all tracks)
12. Visuali–zation and track maps	“method” [step-by-step] “go back over your ‘movie”' “draw” “make a note” “make note on your turn drawings” “marking” “draw yourself pictures of each turn” “Close your eyes and go over the turns in your mind” “Mark down these spots in your diagram” “marking down”	“find out where you don't have enough [reference points]” “listed your barriers”	“the reference points you're not sure of in every turn” “the places you hesitate, go blank, the scene gets foggy or where you hurry through it too fast” “indicates you have too few reference points” “the spots where you're having difficulty of making mistakes” “parts will be foggy, unclear or just not there” “places that are barriers to you, whether they're caused by uncertainty, rushed time, mistakes or other problems”	“use it anytime” “at the track, in between sessions and races”

**Table 3:** Classification of the twelve DPP into the three levels of the McRuer hierarchy, and putative perceptual-cognitive mechanisms that are the target of improvement.

**Level**	**Deliberate practice procedure**		**Perceptual-cognitive capacities targeted for improvement**	
Control	C1	“*Feel*” for grip and traction	The use of **multisensory feedback** to gauge available grip and traction (changes in *friction* and *load* on each tire), esp. under hard braking, steering and acceleration (i.e., in *limit handling*).	Multisensory integration and stabilizing motor routines
	C2	Sense of speed #1	The use of **multisensory feedback** to gauge *speed*.	
	C3	Sense of speed #2	The use of **multisensory feedback** to gauge *speed*.	
	C4	Sense of speed #3	Use of **multisensory feedback** to gauge and fine-tune **motor routines** adjust speed	
	C5	‘*Smooth'* control	Developing **motor routines** with the higher time derivatives of vehicle/controller position (*jerk, snap*) better adapted to vehicle dynamic response.	
Guidance	G1	‘*Looking ahead*'	**Visual strategy:** i. **looking far** enough ahead (but not too far), ii. using as **gaze targets** known **reference points** – *landmarks* or *waypoints* **from long-term memory**, and iii. using **covert visual attention** (peripheral vision) as well as overt **gaze control** to **track multiple** perceptual targets.	Visuospatial attention and predictive gaze strategies
	G2	Situational awareness #1	**Covert visual attention** and **visuospatial short term memory, object tracking**	
	G3	Situational awareness #2	**Covert visual attention** and **visuospatial short term memory, object tracking**	
	G4	Peripheral vision (‘*widescreen*')	**Executive control** of **covert visual attention** (to decouple **gaze control** and **visuospatial attention**).	
Navigation	N1	Determining reference points (‘*finding the apex'*)	Using (memory of) **action-outcomes** as **feedback** for updating **long-term memory** (cognitive maps) with waypoints and landmarks, used at the guidance level as **attentional tracking targets** and **locomotor targets** for optimal curve negotiation.	Self-localization and trajectory planning
	N2	Probing reference point spatial memory with mental imagery #1 (chronometric self-diagnosis of spatial knowledge)	Establishing in **long-term memory** (cognitive maps) a sufficient number or **reference points** (waypoints, landmarks…) for accurate **self-localization** and **motor planning** using **mental imagery**.	
	N3	Probing reference point spatial memory with mental imagery #2 (symbolic self-elicitation of spatial knowledge)	Establishing in **long-term memory** (cognitive maps) a sufficient number or **reference points** (waypoints, landmarks…) for accurate **self-localization** and **motor planning** with the use of symbolic external memory representations.	

### DP1: structure

The first characteristic of DP is that it is an activity explicitly designed (often by a coach) for training purposes. It does not necessarily involve techniques that aim at maximal performance, but techniques that aim at maximal performance gains or learning. The techniques are meant to be used out of competition, often alone as solitary practice between sessions with the coach (see DP4).

Examples of this are “forgetting everything else, especially lap times” (minimum elapsed time is the ultimate goal of cornering technique) or “Even if you almost stop coming up to a turn, then accelerate up to it, that's OK. As long as your entry speed was right you got it.” (Over-slowing and having to accelerate in corner entry as such is a major error in technique, sometimes done by novices - but here it is used in a DP drill designed not to achieve minimum lap time but to practice a component subtask: setting the entry speed exactly right). Also passages such as “intentionally turn into corners later than you think you should” and “late apex at first” refer to techniques where the correct line through a bend is iteratively searched by starting from a certainly wrong (but safe) line, and working up toward a faster (but more risky) line^4^. These types of iterative techniques are particularly well-structured, and they may even be laid out it step-by-step procedures to follow.

Also into DP1 are here categorized descriptions of techniques where the skill or technique is made easier to evaluate by verbalizing it or making it otherwise explicit. Examples of this are “think of the word ‘squeeze”' (when learning smooth throttle and brake application), “actually call out to yourself the amount of traction” (when developing a sense or awareness of the level of adhesion of each tire under dynamic load distribution conditions), or exhortations to “ask yourself” or “ask your brain” diagnostic questions. A particularly interesting subtype of this kind of tasks structured to elicit learning experiences, that is clearly paradigmatic DP, is using mental imagery and self-elicitation of knowledge (drawing track maps), which allow the driver to make explicit and analyze their own long-term memory between driving sessions.

Finally, when the activity is explicitly referred to as “exercise,” “drill,” or “coaching,” or a “method” or “technique” where it clearly does not refer to techniques to directly achieve maximum performance in competition this is interpreted as indicative of structured practice (DP1) in the above sense.

### DP2: goals

The ultimate goal of DP is improved performance, but it often also involves setting clear and explicit subgoals. The entry speed adjustment described above is a case in point. Other examples of practice-session specific subgoals include “reading how much traction the tires have around every inch of the track” or “[becoming] very accurate, and most important, consistent at judging and establishing a specified speed” (facilitated by the verbalization structure mentioned).

A much-discussed driver development goal in the literature is becoming “smooth” (or “progressive” or “gentle”) in operating the controls, which can be practiced in everyday driving as well as on the track. More technical aspects of cornering technique include “determining whether you had the correct apex,” or “the right line” (for which there are iterative procedures given, as mentioned).

Of particular interest with respect to the core perceptual-cognitive expertise are exercises that ask the driver to develop their peripheral vision and covert attention. Here the goal is achieving a kind of “panoramic” or “widescreen” state of vision—where “it doesn't seem like you're looking at anything in particular. The reference points just blend into the scene.” Phenomenologically, the racing driver is given the goal to become “aware of everything.” Likewise, the goal of “looking far enough ahead” and having enough “reference points” in memory that can act as suitable visual targets are interesting goals in terms of the underlying cognitive processes.

### DP3: feedback

The aspect of DP that is probably the most important for skill development (and at the same time perhaps the most neglected in the DP literature) is that the tasks should produce clear and unambiguous feedback. During maximal performance, such as in competition, feedback may be clear (win-lose), but it may be difficult to observe and digest. DP procedures may simplify the task, adjusting task demand to be in balance with current skill, or focus on acquiring and using feedback of just one aspect of the overall task. Coaches or instruction may also point out what the relevant feedback (in the task) is, thus helping the athlete in developing their sensitivity and awareness.

When learning “smooth” controller operation, the racing driver is instructed: “Don't pounce on the gas pedal” “Don't slam on the brakes” Don't yank or jerk the steering wheel “Don't bang the shifter into gear.” This may be interpreted as instructing the driver to observe and be mindful their own actions, and in other words, they are given cues that help them to diagnose errors, and catch themselves doing things that indicate that they are not operating the vehicle in the proper manner; not being “smooth.”

In developing a calibrated sense of speed, the racing driver is asked to accelerate or decelerate to a pre-decided speed, and then check accuracy against speedometer or radar gun readings. In the peripheral vision drill, the driver should keep eyes on a fixed spot in the middle of a wall and introspectively gauge whether they can make themselves “are aware of the other areas”.

In two techniques that help racing drivers to probe the accuracy and detail of spatial knowledge and motor programs in their long-term memory, mental imagery is used to provide a different, more complex kind of feedback. First, by timing their “mental movie” of a lap drivers can use mental chronometry to produce unambiguous feedback (whether running a track in memory takes the same amount of time as the real lap). In another technique, introspective haziness while running one's “movie” indicates areas of uncertainty. These are then written down on a track map, to make explicit the regions where more thought and practice needs to be expended. Both these techniques can be considered ways to create feedback by externalizing information (or the lack of it) in long-term memory.

### DP4: repetition

A defining characteristic of DP is that effort is expended on performing the practice tasks repeatedly. The most salient aspect of the DP literature is the hypothesis concerning the total amount of DP required for world-class expertise: the “10,000 h rule.” This is, of course, not really something that one would consider a thing to look for in DP procedure descriptions (“spend 10,000 h on…”) but, as analyzed in the Introduction, this is not part of the definition of whether some accumulated practice is DP or not—i.e., whether it is on one hand distinct from everyday experience, and on the other hand not aiming for maximal performance.

For the present purposes, repetition was identified in passages such as “do it again and again,” or dedicating “a session” or a substantial number of seat time (laps) to a procedure that does not aim at maximal performance, but rather improvement of specific skills and knowledge (as per DP1-DP3). When techniques are explicitly said to be done “over and over again,” or it is implied that a large volume of practice to build a routine is meant to be amassed out of competition or between sessions where the coach is present—e.g., while driving in “everyday life” or “on the street”—this is classified as DP4.

## Discussion

**Figure 2:**
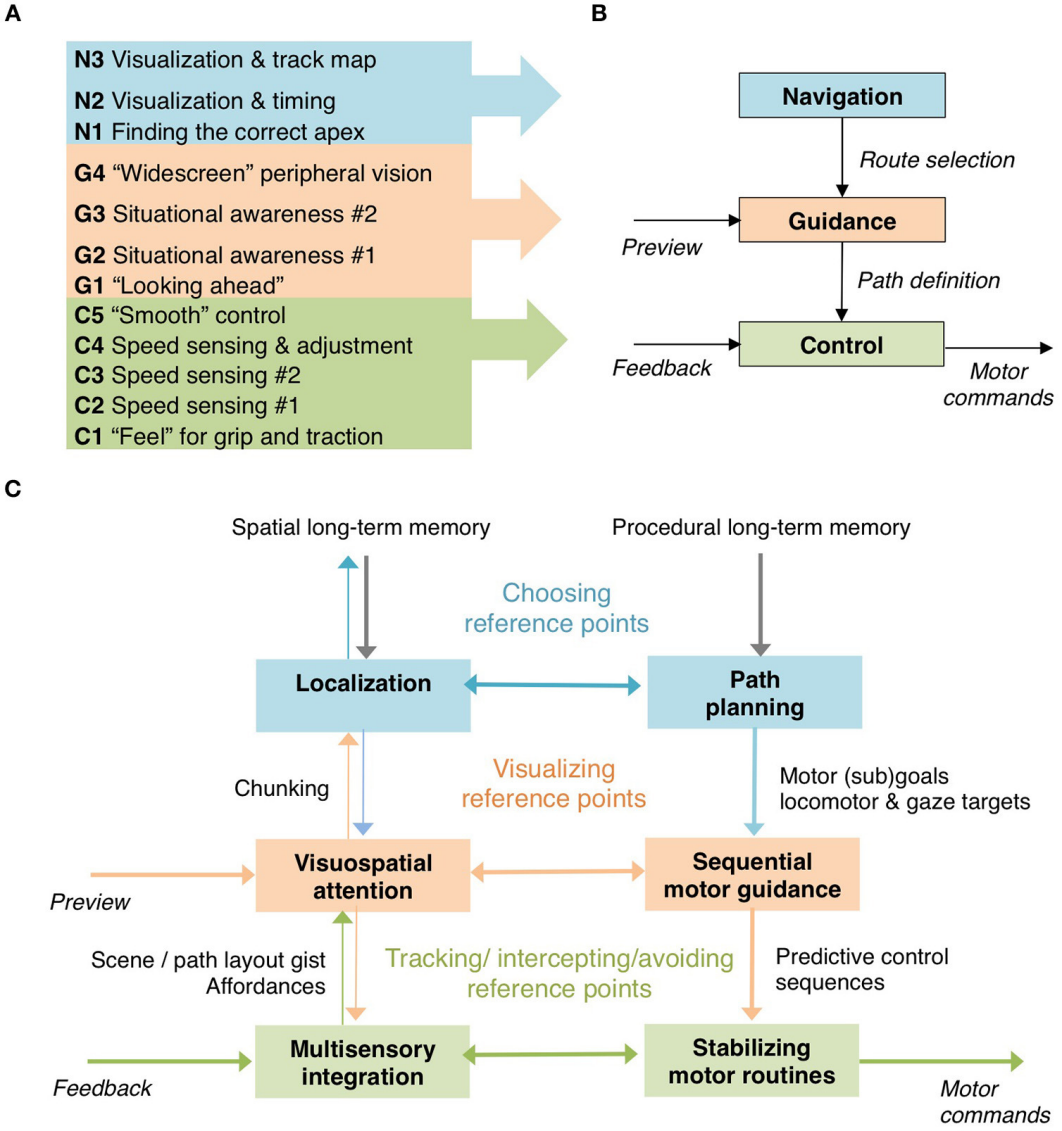
DP procedures **(A)** classified according to the three-level McRuer et al. (1977) wayfinding hierarchy **(B)**, and interpreted **(C)** in terms of putative cognitive processes within and between the levels.

Core-perceptual-cognitive underpinnings of expertise targeted by the DPP are discussed in this section, based. The McRuer conceptual framework is used taxonomically for classifying the DP Procedures to different “levels” of the task (Figure 2A,B). Then, theoretical proposals are made with an eye toward integrating of the picture of expert motor racing emerging from the DP procedures with current psychology and neuroscience.

### Classification of DP procedures in the McRuer hierarchy

Wayfinding tasks, or complex tasks more generally, are understood to have a sequential and hierarchical organization (Lashley, 1951; Cooper and Shallice, 2000; Botvinick, 2008; Jeon, 2014)^5^. Hierarchical organization of the driving task, specifically, is a standard approach in traffic psychology and engineering (McRuer et al., 1977; Donges, 1978; Michon, 1985). The hierarchy proposed by McRuer and co-workers in the 1970s (Figure 2B) has become the de facto basis of design and development of driver models in vehicle system dynamics engineering.

It analyses driving into tasks at three levels: *navigation, guidance*, and *control*. Navigation refers to the selection from among alternatives of the overall route to a goal. At the guidance level, “path definition” is based, *inter alia*, on visual preview information about road/scene layout determining the desired future path; it specifies the control targets to be compared to feedback and effected at the control level. Control refers transformations between feedback and motor commands that implement the decisions made at the higher levels.

The identified DP procedures can be given a natural interpretation as targeting perceptual-cognitive and motor planning processes at different levels of task hierarchy (Figure 2A,B), as follows:

**Control Level DPPs C1-C5** concern sensory feedback and motor commands. C1 develops an accurate feeling for the grip and traction, i.e., the force generating capacity of the tires. This is the foundation of the ability to take a vehicle consistently to “the limit,” and operate it in the regime of nonlinear tire behavior. C2-C4 develop the sense of speed, and the sensorimotor skill in adjusting it with accuracy. C5 concerns fine-tuning one's motor routines for operating the controllers in a “smooth” manner that couples best with the response dynamics of the vehicle. Thus, all have to do with developing *more sensitive and reliable discrimination capabilities for relevant feedback* (information about tire adhesion and lateral forces, vehicle speed); either to enhance processing of naturally occurring feedback, or provide additional feedback to calibrate subjective perception with physical ground truth.

**Guidance Level DPPs G1-G4** are about using visual preview: *visual strategies for active sampling* using eye and head movements, as well as developing *peripheral vision* and “*visualizing*” the path. G1 is a drill for developing visual strategies for sampling the road. G2 and G3-G4 are also about preview, and moment-to-moment path definition - though not only in terms of the fixed 3D scene layout but also taking into account leading or overtaking vehicles. (This kind of “situational awareness” goes beyond current engineering and psychology steering models, which do not discuss car following or overtaking (which are treated as separate phenomena).

**Navigation Level DPPs N1-N3** are about the highest-level route selection tasks. The drills are designed to enhance the development of survey knowledge of the track (“cognitive maps”). N1 is an elementary procedure for explicitly planning ones “line” through a bend. Determining the correct location to apex is the paradigmatic problem solving skill many of the textbooks spend a lot of time explaining. (For an expert this likely becomes highly automatic and intuitive, and may not be analyzable in such detail as in textbook accounts, though). N2 and N3 are visualization exercises. Visualization techniques are used in many sports. N2 asks one to visualize one's performance (from an egocentric perspective) and to indicate on a map (allocentric perspective) important visual targets, as well as areas where the visualization is “hazy”—as this indicates areas of the track where the memory/action plan are unclear and more work is needed. N4 is interesting in that it uses mental chronometry to probe the *accuracy* of memory/action plans (by comparing lap visualization duration to actual lap times). Expert performance in all fields is founded on vast reserves of long-term memory that can be called upon. These procedures therefore are at the heart of expertise as they probe and develop one's long-term memory structures and make them explicit.

### Proposals for integrative hypotheses of core perceptual-cognitive mechanisms

A perhaps striking feature of the DP procedures is their variety - in particular how “high level” cognitive processes are involved. While some drills target the “feel” for speed and the balance of the vehicle—presumably largely automatic and unconscious processes—others involve explicit memory recall, visualization and problem solving.

Hence, to get to the root of expertise in this domain, it is clear there is a need to understand more than sensorimotor (eye-hand) coordination, or basic sensorimotor transformations. We will start from the higher-level processes, furthest removed from the directly observable sensorimotor periphery. This will actually introduce key concepts that will become useful as we work our way down toward the most basic sensorimotor skills, the foundation of domain expertise and closest to the more extensively studied and understood driver-vehicle system dynamics.

#### Navigation mechanisms: self-localization and path planning

The track map and imagery drills (N3 and N2 probing long-term memory and mental imagery by track maps and chronometry, respectively) rest on processes at the top level of wayfinding hierarchy. They relate to cognitive processes such as *localization* (cognitive maps) and *path planning* (generation of complex action goals for eye-hand-body movement sequences, using internal models).

Racing drivers are taught techniques to “fix” the track in their minds, so that they can visualize driving it in great detail. It is currently unknown is what the “mapped” elements are—i.e., what counts as a landmark or a reference point the racing driver uses to localize themselves. Visualization techniques are also used to self-evaluate long-term memory for track locations, landmarks and action sequences—and the way they are combined, i.e., action plans. Locations where the track layout is not yet “fixed,” or where the appropriate action is not clear, will be experienced as “hazy” imagery, or excessively fast or slow “mental replay,” and will leave blank areas in a track map if the driver is asked to depict his or her landmarks and actions in a schematic map of the circuit.

These DP procedures probe landmark and reference point information in long term memory—what may be termed cognitive maps. Note that the term cognitive map is not used here to denote any specific theoretical assumption about the long-term memory involved in these tasks—e.g., whether the relevant knowledge is represented in egocentric or allocentric reference frames, to what extent it is metrically accurate, or to what extent is it globally consistent and truly “map like” vs. “graph like” with only limited topological constrains (cf. Chrastil and Warren, 2014, 2015; for theoretical discussion see Meilinger, 2008). This issue directly relates to fundamental theoretical issues in human wayfinding, an active field of research in basic neuroscience (for recent reviews see Epstein and Vass, 2013; Spiers and Barry, 2015; Epstein et al., 2017).

But whatever the detailed format and content of the relevant long-term memory turns out to be, the drill N2 (visualization & timing) in particular suggests, interestingly, that action planning (even at the level of timing) is very closely coupled to such localization level spatial memory read-out. Further understanding of this phenomenon would be novel and theoretically and practically important^6^.

From a cognitive point of view, it is perhaps not surprising or theoretically controversial that a large body of knowledge in long-term memory is involved. This is after all the case of all forms of expertise studied to date. But perhaps more debatable is the relation of this vast body of knowledge to online performance. N2 and N3 are after all purely offline drills. They are not done while performing the driving task. Understanding the interplay of explicit offline knowledge and implicit visuomotor skill has implications for the psychology of expert performance and training - in particular the current theoretical discussions over whether representational, cognitively mediated processes are involved, or whether automatic skill is achieved in a purely embodied, situational way through action mediated by dynamic interactions with the physical environment (Dreyfus, 2002; Wilson and Golonka, 2013; Zhao and Warren, 2015).

A fact beyond serious dispute is, however, that a race track simply cannot be negotiated at high speed on the basis of just the visual appearance of the bends—“the line” *must* be based on the recall of stored knowledge of the scene layout of the particular track beyond current field of view.

Perhaps the most paradigmatic textbook DP procedure is N1 for finding the correct line in a bend or a series of bends, on basis of determining the correct location for the apex. Fixing this locomotor (sub)goal is presented as a quite systematic search procedure: first *changing* the decision about the location of (a reference point at) the apex one “aims for,” and then *observing* vehicle behavior at the curve exit—i.e., how early and hard one was able to open the throttle, how tight a line one was still able to take when exiting the bend, what speed the exit of the bend was reached—and then *adjusting* the position of the apex to find a faster line.

Theoretically important in terms of basic cognitive processes here is that carrying out this procedure requires very accurate episodic short-term memory recall of where and when in the bend what specific actions were taken, and how they affected the perceived performance outcomes (elapsed time, exit speed, vehicle understeer/oversteer…). That is, in order to evaluate and adjust “the line” for each individual bend on the following lap, this must be based on memory of the *preceding* run through it. And here the racing driver does not rely only on a general gut feeling - for example “I should try harder” or “I should ease off”—but on a much more detailed and fairly detailed analytic memory of specific aspects of performance during the previous encounter.

The apex-finding drill also introduces the pivotal concept of reference points (brake markers, apex waypoints, gearshift timing points at the exit etc.…). This may give more clues as to what the underlying memory representation is like. One possibility is that a “library” of stereotypical curve templates is stored in long-term memory—analogous to familiar (sub)patterns of chess pieces—and that the racing driver draws upon these when presented with a new situation. This would allow the experienced racing driver to quickly “chunk” for memory encoding and recall complex but familiar track layouts into reference point configurations, overcoming short-term visual and working memory capacity limitations (cf. Chase and Simon, 1973a,b; Simon, 1974; Glaser, 1985; Gobet et al., 2001, 2016; Gilchrist and Cowan, 2012). I thus propose that, at the higher levels of wayfinding it is precisely this kind of *chunking* of the scene in terms of familiar (sub)patterns of reference points overlaid on a scene layout (the lower-level *affordances* or *gist*) that is the core of perceptual cognitive expertise of the racing driver. In further speculation, when it comes to “learning a track,” could the prefrontal–parietal–retrosplenial–hippocampal network, which is implicated in the neural substratum of racing expertise (Bernardi et al., 2014), be involved in just such hierarchical “chunking of the driving line” in terms of oculomotor/locomotor subgoals (Lappi, 2015; cf. Cooper and Shallice, 2000; Jeon, 2014)?

In various experimental tasks, and some domains such as chess, theories of chunking have been for decades the default approach to understanding human skill acquisition and expertise (Glaser, 1985; Rosenbloom et al., 1987; Gobet et al., 2001). Yet the concept of chunking appears not to have been heretofore applied in the domain of expert driving, and on the whole, how athletes generally (not just racing drivers) chunk spatial knowledge and motor sequences into meaningful higher-order patterns is still a very much unexplored subject.

#### Guidance mechanisms: dynamic visuospatial attention and predictive motor sequences

Reference points are “known locations” on the track. This means that—one way or another—they enable self-localization and orientation in terms of a cognitive map in long.term memory. But for any of this information to be of use in actual performance they need to fit seamlessly into the flow of online action control. How they are probed, or perhaps manipulated, in memory and decision-making processes offline gives limited understanding of how this is achieved. We must also understand *dynamic visuospatial attention* and *predictive motor sequence generation* blend top-down (localization, path planning) and bottom-up (multisensory perceptual feedback, motor commands) processes. Can the drills at guidance level suggest hypotheses about how this integration may be achieved?

One possibility is that reference points are used as waypoints in some form of “looking where you are going,” “going where you are looking,” or “looking where you want to go” visual strategy (Wann and Swapp, 2000; Wilkie et al., 2008; Lappi, 2014; Lappi and Mole, 2018). By waypoints are meant fixed locations on the track that are simultaneously gaze targets (locations the racing driver looks at) and locomotor targets (locations the racing driver steers to pass through), one subtype of reference point.

Indeed, G1 (looking ahead) explicitly talks about seeking (suitably far ahead) reference points as gaze targets. However, while the waypoint hypothesis may hold for some reference points, looking at the other DP procedures related to visual strategies and visuospatial attention suggests this may not be the whole story. G2 and G3 involve training ones' capability for simultaneously monitoring multiple locations and objects on extrapersonal space—one's Situational Awareness (cf. Endsley, 1995; Smith and Hancock, 1995). Clearly, the majority of these will *not* be waypoints. In terms of basic cognitive processes, at least multiple target *tracking*—extending beyond the current field of view—and *predicting* their path seem to be implicated.

These processes are intertwined with the role of *peripheral vision* in guidance. For understanding the complementary roles of gaze strategy (overt, focal attention) and peripheral vision (covert focal attention and ambient visual processing) perhaps the most interesting guidance drill is G4 (“widescreen” peripheral vision). This is a drill for developing covert attention by using *executive control* to deliberately decouple gaze direction and the direction of visuospatial attention (a fairly complex and not intuitively natural goal). Its rationale is, according to the literature, training the right way of “looking” at the race track: using peripheral vision to be “aware of everything” (Bentley, 2003, p. 50). As for waypoints, this suggests that the reference points may not be best described as singular “steering points” (see Lappi, 2014 for a review of steering point literature in psychology), but more complex configurations of reference points in the 3D scene forming recognizable patterns that are “taken in” partly through peripheral vision (that is: chunks).

However, as a cautionary word it needs to be pointed out that people, including experts, typically have quite poor insight into one's own gaze behavior (Burr and Morrone, 2012 report data from an art painter who likewise claimed to “take it all in” in one glance, but when actually measured displayed extensive saccadic scanning when perusing her own work). Experimentally, there is still little quantitative understanding of the actual gaze strategies of expert race drivers (though see Land and Tatler, 2001; van Leeuwen et al., 2017).

#### Control mechanisms: multisensory integration and stabilizing motor routines

Sensorimotor transformation processes are brain mechanisms responsible for coordinating how information from perceptual processing and feedback signals is used to control motor commands. They determine at the finest level of detail amenable to “cognitive” analysis by just how much, exactly how fast and with precisely how much force the racing driver will operate the vehicle controls in any given situation. It may intuitively appear the most straightforward of the levels to understand - but in fact the “unconscious automaticity” of skill (and possibly also comparison with the comparatively trivial task of everyday driving which, phenomenologically at least, may appear almost devoid of a “cognitive component,” cf. Dreyfus, 2002) belies a remarkable sophistication and complexity in the information processing required. (This clearly revealed in artificial intelligence and robotics).

In a dynamically complex and only intermittently and partially observable task environment, such as a racetrack is, adaptive behavior requires “anticipatory” motor sequences rather than just “good reactions.” Even at the Control level an expert racing driver will likely have developed highly detailed *internal models* (both *inverse* and *forward* models) about the dynamics of his or her body and the vehicle (cf. Miall and Wolpert, 1996; Wolpert and Kawato, 1998; Wolpert and Ghahramani, 2000; see also Keen and Cole, 2006; Wolpert et al., 2011; Engström et al., 2018; Lappi and Mole, 2018). Such internal representations are needed to take skilled advantage of the dynamic dependencies between the controls and action outcomes—especially the complexity of how controls interact, allowing the skilled racing driver to “blend” braking and turning, throttle and gear change etc. in a “smooth” way. This is known as having a good “feel” for or skill in “balancing” the vehicle “on the edge.” In light of this, how can we interpret the Control level drills in terms of basic perceptual-cognitive processes targeted?

C1 (“feel”) is about developing the necessary very fine judgment of the available grip and traction, and how they are affected by track conditions and vehicle responses to control inputs (especially under hard braking, steering and acceleration). This “feel” is perhaps the most basic skill, and arguably the foundation on which all other skill and “talent” is based. What is the perceptual-cognitive basis of “feel”? The racing driver cannot directly observe either the coefficient of friction or vertical load on a tire. Instead the grip must be estimated from changes in steering wheel self-aligning torque, somatosensory and haptic feedback through the seat of the pants, sound (from the tires, engine and wind noise), vestibular signals (acceleration, especially rotational acceleration in the yaw plane) and visual idiothetic cues (such as optic flow)^7^. This is a highly complicated *multisensory integration* problem. What is more, not only does each tire have a different instantaneous grip level, but it is really the changing patterns of grip, based on successive states of load distribution^8^ that create the “handling balance” of the vehicle, which the driver must, in order to produce fast and consistent lap times, have a very accurate “feeling for.”

In order to produce predictable vehicle behavior, control must be based on an ability to anticipate future grip traction (presumably based on intended control actions and past observations of control action outcomes). The real problem for the racer's brain, then, is whether friction on the road ahead and *imminent* rather than *current* load distribution will allow specific steering wheel, throttle or brake input sequences (at a given speed)^9^. Grip judgment is thus not based on (delayed) feedback only, but needs to be based on top-down information from internalized forward models of vehicle dynamics, and inverse models determining upcoming motor actions (e.g., Cole et al., 2006; Keen and Cole, 2006, 2011). So, although in some respects the most basic skill, in fact “feel” and “balance” are maybe the one of the most complex to analyze, as it is at such a minute level of detail that it needs to be understood to understand the skill underlying practically important performance differences.

In contrast, the visual control of steering in experimental psychology are very simple *steering point* models (e.g., Land, 1998; Salvucci and Gray, 2004; for review see Lappi, 2014), yoking single control output directly to feedback. In practice, these models are required only to account for very modest (novice) performance in simplified laboratory experiments or at most simulated everyday driving tasks. These are dynamically trivial compared to actual race driving, allowing the field to make do with very simple control models. On the other hand, vehicle system dynamics engineering requires its steering models to achieve high-level limit handling performance (for driver-in-the-loop simulation; for review see Macadam, 2003). This field has turned to predictive coding and paired inverse and forward models from artificial intelligence and robotics (for discussion of the relation of such models to psychological driver models and neuroscience see Nash et al., 2016; Lappi and Mole, 2018).

C2-C4 (sense of speed), seen from a cognitive perspective, also has to do with multisensory integration. Vision (especially optic flow, also speedometer and crankshaft rotational speed gauge) hearing (engine, wind, tire noise), somatosensation (vibrotactile sensations from the engine and chassis) and linear and rotational acceleration sensed via the inner ear are likely integrated to estimate self-motion. In comparison to “feel” this process is more straightforward to analyze as only the relatively simple physical variable of speed is involved—at least when constant speed in a straight line^10^ is concerned. (It is nevertheless much more complex than most laboratory psychology tasks that are usually based on only one modality).

C5 (“smooth” control) is about finessing skills on the motor end of control: motor routines for the proper use of steering throttle and pedals. The term “smooth” itself is somewhat difficult to interpret. For one thing, seems to imply stabilization, not “upsetting the balance,” and also a certain economy of movement. “Smooth” is at the same time progressive and decisive (as opposed to hesitant; e.g., “once throttle is cracked open it is opened progressively”). But decisive here does not mean brutal (e.g., the speed of gear change and the timing of clutch and throttle use needs to adapt to vehicle drive train). “Smooth” seems to imply what is often called “mechanical sympathy.” Finally, smooth and progressive does not mean slow, the controls are operated quickly—but it means the controllers should be moved only as fast the vehicle will “take,” and as fast as the driver can “stay on top of.” What is thus likely important here is that the motor routines are such that (i) the timing and amplitude of controller movement are predictable, (ii) that they match the response rates of the vehicle, and (iii) motor routines for steering and brake/throttle control produce appropriate higher derivatives of position (acceleration, jerk, snap) of the controllers and vehicle components. Such *stabilizing motor routines*—a putative definition for being “smooth” with the controls—may contribute also to the accurate sense of dynamic balance (“feel” discussed above), as the vehicle responses themselves become more predictable and progressive. This hypothesis implies that there exists a tight coupling between perception and action: a good “feel” for the vehicle aids in “smooth” control, but “smooth” control also aids in state estimation as the state transitions become less abrupt and random.

### Discussion summary

To bring things together, Table 3 dissects the core perceptual-cognitive expertise of the racing driver—or aspects of it revealed by analysis of Deliberate Practice Procedures found in the professional motor sport coaching literature—into basic cognitive-perceptual components. Figure 2C in turn indicates in rough outline the relationships of the above to one another, and the McRuer hierarchy.

The key theoretical proposal of this paper is that the discussion of reference points used for guidance, driving “the line” and navigational memory for track locations be interpreted in terms of chunking. This proposal is motivated by a substantial body of experimental and theoretical work on perceptual-cognitive expertise in a number of domains. In now-classic studies on chess (Chase and Simon, 1973a,b; deGroot, 1978), experts were shown to be superior in quickly encoding complex relational information (recall of game positions after a brief exposure was much superior to novices, an advantage that was negated by removing relational information by scrambling the pieces to random positions). This research directly led to the development of the chunking theory of expert perception and memory. According to this theory, rather than more extensive generation of and search among choice alternatives experts' superior performance rests more on pattern recognition (“chunking” positions into a few sets of meaningfully related items) driven by information stored in LTM. In addition to memory recall and decision making, process tracing methods such as verbal protocol analysis and eye tracking (Charness et al., 2001) have been used to understand this knowledge representation underlying encoding and retrieval. These methods have been also used to analyze encoding relational information in dynamic scenes in sports where the relevant “items” (i.e., players) are similarly spread out in space, such as football (Williams et al., 2006; Poplu et al., 2008; North et al., 2009, 2011; Roca et al., 2011; for work on contextual information beyond kinetmatic relations cf. also Cañal-Bruland and Mann, 2015; Mann and Savelsbergh, 2015; Murphy et al., 2016). There, relational information (the geometrical positioning of players and the ball in the field, analogous to placement of pieces on a chess board) has been shown as meaningful for domain experts.

The present proposal, then, is that 3D scene structure is similarly “chunked” as relational information: a pattern of items (reference points) in a specific arrangement, such that items within a chunk strongly associated with one another and specific procedural knowledge, and more loosely associated with items and procedural knowledge of other chunks. It should be noted that this analogy between reference points and chess pieces/players is a radically different view of the underlying knowledge representation from what is posited in computational steering models, which represent the information extracted from scene preview as either a “desired path,” or one or two “steering points” (for review see Lappi, 2014; Lappi and Mole, 2018). Thus, it the present proposal is on the right track, while such algorithmic models may replicate some aspects of task performance they do this on the basis of different representation than used by the human.

Further analysis of the component tasks and operationalization of key concepts (such as reference points) in simplified but more controlled experimental settings should, in the future, further elucidate the processes for perceptual encoding and memory retrieval, and help develop more ecologically valid and representative experimental designs to investigate expert performance in this domain. It should also allow more systematic relations to be defined between *engineering* concepts (and related *racing jargon*), *neuroscience* and *experimental psychology*, and computational concepts *in cognitive science*. This will help bring experimental work on behavior and brain function to bear on the psychology of the racing driver, so that, in time, this critical component of the driver-vehicle system might be understood in more detail.

## Conclusions

In light of DPP in motorsport, what are the core perceptual-cognitive basis of expert-level high-speed locomotor performance? While fine individual differences in “feel” and skill in “smoothness” are probably important factors for differentiating experts at the higher (elite) level, the present analysis suggests that, like all domains of expertise to date, also racing performance is dependent on a vast base of task relevant domain knowledge. The racing driver's expertise involves, and may be based on long-term memory processes to an extent that is perhaps surprising from the point of view of current driver modeling state-of-the-art (less so from the point of view of the cognitive psychology of expertise).

How is the relevant knowledge organized? How is it applied in real time in the highly time-constrained, dynamical task? The relevant domain knowledge may, according to the present proposal, take the form chunking bends into prototypical patterns of reference points (landmarks, waypoints, timing cues), localized and tracked in psychological space. This view is well in line with the most general finding in 40 years of research into expertise—that superior performance of experts (compared to novices) is based on the large amount of domain knowledge and the qualitatively different way it is structured—but quite novel in the driving literature.

This means that while for everyday driving the driving task could perhaps be represented essentially as simple path following, for racing experts “the line” would instead be based on general principles underlying pattern recognition, memory, and action selection similar to other domains of skill and expertise studied in cognitive science. At the expert level, steering a series of bends cannot be reduced to a simple visuomotor routine: the experts' detailed survey knowledge of the track and a deep understanding of cornering techniques may bring into play many cognitive operations not found in “merely experienced” everyday driving.

In order to design representative tasks for experimental psychological or neuroscience investigation, or to interpret pattern in experimental (e.g., eye tracking), or observational (e.g., driver-coach interaction) data, it is necessary for the field to develop a deeper domain understanding—i.e., sound and detailed analysis of the cognitive task demands placed on the racing driver going beyond “following the desired trajectory” based on vision and memory. Also, the lack of detail in behavioral research limits the chances for integrating the computational driver-in-the-loop modeling in engineering (Sharp et al., 2000; Macadam, 2003; Keen and Cole, 2006, 2011; Sharp and Peng, 2011; Nash and Cole, 2018) and experimental work in psychological and visual science research (cf. discussion in Lappi and Mole, 2018).

New modeling ideas are needed, and this in turn requires data from experimental tasks representative of expert performance, which would allow the skill components to be isolated and investigated. Development of such new paradigms may be facilitated by domain insight that in turn can be gleaned from knowledge elicitation methods, such as qualitative document analysis as employed here.

It should be noted that the qualitative approach can serve to develop hypotheses about the processes underlying domain expertise and *ground* them in elicited expert knowledge. It cannot *empirically establish* the hypotheses. Rather, knowledge elicitation serves the important heuristic purpose to foster the development of well-grounded research questions and experimental designs. The empirical validity of any emerging hypotheses must eventually be investigated experimentally. It is the hope that this qualitative research paper may contribute to quantitative experimental work that will yield a deeper understanding of this under-researched domain of sports expertise - which also happens to be in many ways an ideal model system for studying fundamental issues of spatial cognition and expert performance it supports.

## Data availability statement

The analyzed dataset for this study can be found on Figshare: https://doi.org/10.6084/m9.figshare.6078632

## Author contributions

The author confirms being the sole contributor of this work and approved it for publication.

### Conflict of interest statement

The author declares that the research was conducted in the absence of any commercial or financial relationships that could be construed as a potential conflict of interest.

## Footnotes

^1^In comparison to experienced non-expert drivers, racing drivers' gaze seems to be less focused on the apex (or tangent point) region and to show more variability and anticipation (Land and Tatler, 2001; van Leeuwen et al., 2017). But the precise quantitative characteristics and theoretical nature of this “variability and anticipation” remain to be defined.

^2^This aspect of acquiring the necessary domain insight seems under-used or at least under-reported in experimental psychology where experiments are commonly designed on the basis of experimenter intuitions or domain expert advice, but the process is typically left undocumented. A more systematic approach to task analysis and design would seem desirable.

^3^Not all experience is equal—accumulation of “mere experience” does not dramatically improve performance in the way Deliberate Practice will. Thus, one can have 10,000 h of experience in some activity—such as driving—but this does not make one an expert, if the experience is just “more of the same.” This is likely the case for driving in everyday traffic for most people most of the time, where the goal is satisficing task demands (Summala, 2007). In contrast, constant self-improvement—optimizing performance—requires effort of a kind characteristic of DP. Hence, the study of highly *experienced* drivers' perceptual-cognitive skill is likely relatively uninformative for understanding *expert* racing driver performance.

^4^The *apex* or “clipping point” is the part on the track where the vehicle trajectory cuts closest to the inside of the bend. The location of the apex largely determines the timing, localization and amplitude of braking and steering input needed to adjust speed and turn into the bend, and the location is not determined by geometry. The driver chooses “where they want to (clip the) apex.” This is usually not at the geometrical mid-point or point of highest bend curvature - instead the optimal placing of the apex point depends on vehicle characteristics, longitudinal and lateral elevation changes and whether the bend is preceded or followed by straights or other bends. A “late” apex sacrifices some speed entering the bend but allows the racing driver to accelerate earlier, and there is less risk of running out of road on the exits. An “early” apex is a typical beginner error, the consequence of which is that while the beginning of the bend can be taken at a safer-feeling large radius arc, the driver arrives at the clipping point at a high speed and the vehicle pointing towards the outside of the bend. They will need to tighten the line, which slows the vehicle down and may lead easily to loss of control (spin), because weight is shifted off the rear axle.

^5^“Hierarchical” is here taken to mean that (i) high level behaviors are composed from lower level actions performed in a specific order, (ii) higher-level behavior is thereby more extended in time, and (iii) higher level goals may be achieved by various lower level actions. Higher-level cognitive processes *may* also involve more “abstract” information processing, and/or rely on neural circuits further removed from the sensorimotor periphery, but this should not be taken as definitional.

^6^Assuming, of course, that the endogenous timing is as accurate as the authors suggest, and that there exists a correlation between this task and driving performance. Neither fact has been experimentally determined.

^7^The somatosensory body schema is known to be quite plastic and modifiable with tool use, so the vehicle may even be considered in a literal sense an extension of the driver's body (or peripersonal space). How the body-external space divisions of “near”/peripersonal up to the body boundaries and “far”/extrapersonal space map into neural representations, and the plasticity of this mapping has been mainly studied in the domain of reaching, grasping and tool use (see Berti and Frasinetti, 2000; Maravita et al., 2001), but little investigated in the context of locomotor behavior. (Though see discussion in Trevarthen, 1968 and Previc, 1998).

^8^Actually the instantaneous grip and traction offered by a tire will depend in a complex way on a much larger number of interdependent factors than just the vertical load on the tire and the coefficient of friction at each contact patch. These additional factors include: the longitudinal and lateral inclines of the road, the tire's inherent load sensitivity, the orientation of the tire relative to the direction of travel and the vertical (slip camber angles, respectively), the size of the contact patch, tire pressure, tire stiffness (itself dependent on construction and operating temperature), vehicle speed and tire rotational speed and their ratio, tire compound, and contact patch temperatures). See Milliken and Milliken (1995) for details. It is not known to which of these factors and expert racing driver is particularly sensitive.

^9^Grip “used up” by lateral acceleration cannot be used to accelerate or decelerate the vehicle, and vice versa. Also of note is that when speed and direction vary over time and there is lateral and longitudinal weight transfer, all the controls generally affect *both* lateral and longitudinal control. Only in the idealized case of constant speed can lateral control (steering) be equated with turning of the steering wheel.

^10^Especially in curves the subjective perception of speed may well be mixed up with judging the oversteer/understeer characteristics and roadholding of the vehicle, sensed in part through steering self-aligning torque and rotational rate response. That is, a relatively more uncontrollable and unstable vehicle may be experienced as going faster than a more stable and controllable vehicle at the same speed.
